# Berberine: Ins and outs of a nature-made PCSK9 inhibitor

**DOI:** 10.17179/excli2022-5234

**Published:** 2022-08-17

**Authors:** Sarina Ataei, Prashant Kesharwani, Amirhossein Sahebkar

**Affiliations:** 1 Applied Biomedical Research Center, Mashhad University of Medical Sciences, Mashhad, Iran; 2 Department of Pharmaceutics, School of Pharmaceutical Education and Research, Jamia Hamdard, New Delhi, India; 3 Biotechnology Research Center, Pharmaceutical Technology Institute, Mashhad University of Medical Sciences, Mashhad, Iran; 4 School of Medicine, The University of Western Australia, Perth, Australia; 5 Department of Biotechnology, School of Pharmacy, Mashhad University of Medical Sciences, Mashhad, Iran

**Keywords:** berberine, PCSK9, hypercholesterolemia, phytomedicine, dyslipidemia

## Abstract

Proprotein convertase subtilisin/kexin type 9 (PCSK9) is a secreted protein and a critical regulator of low-density lipoprotein-cholesterol (LDL-C) through inducing degradation of the LDL receptor (LDLR) within the hepatocyte lysosome. PCSK9 deficiency significantly improves the survival rate of cardiovascular disease (CVDs) patients. Up to now, various PCSK9 inhibition approaches have been tested. However, the currently available PCSK9 inhibitors' widespread use is limited due to their inconvenient method of administration and high cost. On the other hand, inhibiting PCSK9 with nutraceuticals is safe and affordable. The plant-derived compound berberine has shown anti-PCSK9 activity in several studies. Berberine is an isoquinoline quaternary alkaloid of phyto origin. Berberine treatment boosts the hepatic expression of LDLRs, while decreasing the expression and secretion of the LDLR modulator PCSK9. The current review presents a collection of *in vitro *and* in vivo* studies investigating berberine's effects on PCSK9 mRNA expression, protein level, and function.

## Introduction

Proprotein convertase subtilisin/kexin type 9 (PCSK9) is a key regulator of low-density lipoprotein cholesterol (LDL-C). PCSK9 is mainly expressed by hepatic cells and secreted into the bloodstream. Following its discovery in 2003, it has been studied extensively in cholesterol homeostasis and cardiovascular biology (Seidah et al., 2014[52]). PCSK9's role in cholesterol metabolism was recognized in two French families with the clinical phenotype of dominant autosomal familial hypercholesterolemia and mutations in the PCSK9 gene encoding the protein PCSK9, not previously associated with cholesterol metabolism. With this discovery, interest in PCSK9 protein began to grow, and it is now known to be a highly polymorphic gene (Ascaso, 2016[2]).

**Figure 1:**
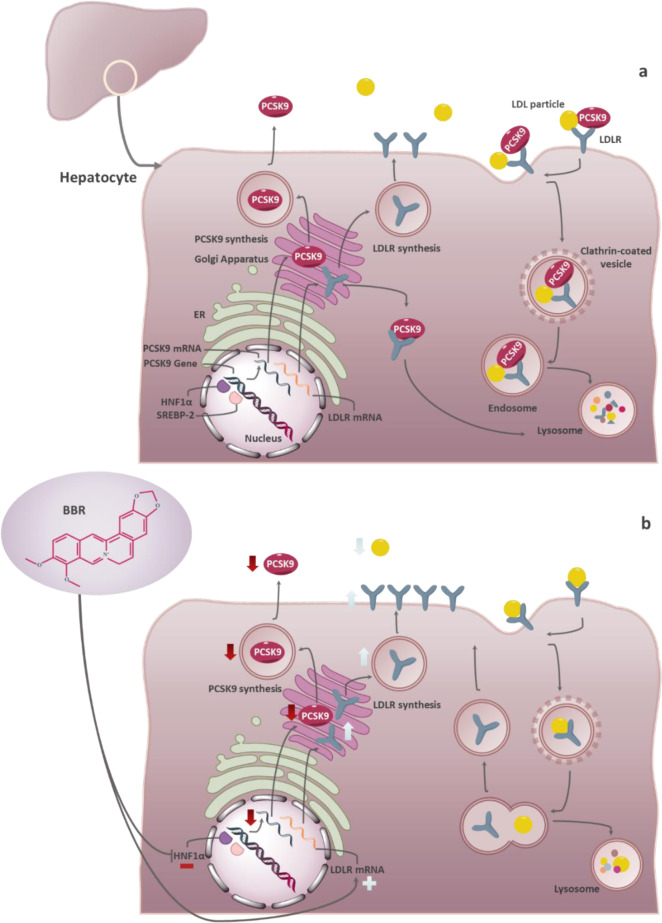
PCSK9 functions with or without presence of berberine. a. PCSK9 mechanism of action. PCSK9 is secreted from the endoplasmic reticulum to the Golgi apparatus and subsequently from the trans-Golgi network into the medium. A secreted form of PCSK9 binds to LDLR on the hepatocyte surface and facilitates degradation of the PCSK9-LDLR-LDL complex (extracellular pathway); PCSK9 directly binds to the newly generated LDLR in the trans-Golgi network, where the PCSK9-LDLR complex is directed to the lysosome (intracellular pathway). b. PCSK9 mechanism of action, in the presence of berberine. Berberine directly facilitates the stabilization of LDLR mRNA. It inhibits hepatocyte nuclear factor 1 alpha (HNF1α) protein, thereby decreasing PCSK9 expression, promoting the expression of LDLR, and limiting its degradation, resulting in more significant clearance of LDL.

PCSK9 is most well known for its effect on low-density lipoprotein receptor (LDLR) in hepatocytes, representing the primary pathway by which LDL-C is cleared from the bloodstream (Peterson et al., 2008[42]; Zhang et al., 2007[64]). In the intracellular pathway, which is comparatively faster, PCSK9 binds to the EGF-A domain of the newly generated LDLR in the trans-Golgi network, where the PCSK9-LDLR complex is directed to the lysosome (Poirier et al., 2009[44]). In the extracellular pathway, the secreted PCSK9 binds to the LDLR's EGF-A domain on the surface of hepatocytes and chaperones it to the lysosome via clathrin-mediated endocytosis (Peterson et al., 2008[42]; Qian et al., 2007[45]) (Figure 1a(Fig. 1)). Since normal recycling of LDLR to the hepatocyte surface depends on the EGF-A domain, PCSK9's binding to this domain inhibits the LDLR from being recycled to the cell surface and instead promotes lysosomal degradation of the LDLR (Atar et al., 2022[3]). It is possible for LDLRs not bound to PCSK9 to be recirculated more than 100 times (Atar et al., 2022[3]). As a result of PCSK9 activity, the LDL-C removal from the bloodstream by LDLRs is reduced, and the vulnerability to atherosclerotic cardiovascular diseases (ACVDs) is increased (Peterson et al., 2008[42]; Zhang et al., 2007[64]). Decreasing LDLR by PCSK9 leads to hyperlipidemia associated with numerous cardiovascular complications. Particularly, macrophages' uptake and accumulation of lipids in the vessel wall and the formation of foamy macrophages contribute to the development of atheromas (Sundararaman et al., 2021[56]). Clinically, PCSK9 deficiency significantly improves the survival rate of cardiovascular disease (CVD) patients (Benn et al., 2010[7]; Cohen et al., 2005[14], 2006[15]). In light of PCSK9's importance in regulating LDL-C and LDLR metabolism and the safety of inhibiting it, PCSK9 has gained interest as a therapeutic target (Catapano and Papadopoulos, 2013[10]). Through PCSK9 pharmacological inhibition, the LDLR action would be prolonged, improving LDL-C clearance and lowering plasma LDL-C levels (Costet et al., 2008[16]).

## PCSK9 Inhibitors and Their Current Limitations

Various approaches have been developed to inhibit PCSK9, including monoclonal antibodies (mAbs), gene-silencing or editing technologies, such as antisense oligonucleotides, and small interfering RNA (siRNA)s; small-molecule inhibitors; clustered regularly interspaced short palindromic repeats (CRISPR)/Cas9 platform; modified binding proteins such as adnectins; mimetic peptides; and vaccination (Ito and Santos, 2017[28]; Nishikido and Ray, 2019[40]). The most extensively studied approach is the mAbs that target plasma PCSK9 and block PCSK9/LDLR interaction. Studies have shown that inhibition of PCSK9 by monoclonal antibodies significantly decreases LDL-C levels and reduces CVD incidence (Catapano and Papadopoulos, 2013[10]; Do et al., 2013[20]; Sahebkar and Watts, 2013[50]). Therefore far, two monoclonal antibodies (alirocumab and evolocumab) have been approved by European Medicines Agency (EMA) and Food and Drug Administration (FDA) for treating patients with hypercholesterolemia in 2015 and have been successfully used in clinics alone or combined with statin (Dixon et al., 2019[19]; Kereiakes et al., 2015[32]; Merćep et al., 2022[39]; Raal et al., 2015[46]; Sabatine et al., 2015[48]). Statins are the most widely administered cholesterol-lowering agents with several pleiotropic effects ([Bahrami et al., 2018[5] Bland et al., 2022[8]; Dehnavi et al., 2021[18]; Khalifeh et al., 2021[33]; Shakour et al., 2020[54]; Vahedian-Azimi et al., 2021[61]]. These drugs act through increasing the expression of hepatic LDLR while concurrently increasing PCSK9 expression, suggesting that their effect on PCSK9 may decrease the therapeutic effect of statins (Sahebkar et al., 2015[49]). Moreover, inclisiran, a novel siRNA-based PCSK9 silencer, has recently been approved by the EMA and FDA and has been proven to reduce LDL-C among patients at high risk of cardiovascular diseases (Cicero et al., 2022[13]; Ray et al., 2017[47]). siRNAs target intracellular PCSK9 (Ochin and Garelnabi, 2018[41]). However, the administration method for these two classes of therapeutics is via the parenteral route, which is inconvenient and limits their widespread use, especially considering the chronicity of LDL-C-associated ACVD (Ochin and Garelnabi, 2018[41]). This feature could make it difficult for patients to comply with treatment. Moreover, the treatment procedure of administering anti-PCSK9 antibodies has several other limitations. Including the short half-life of mAbs *in vivo*, neutralizing antibody production, and requiring multiple injections at high doses may impose a psychological and financial burden on patients (Galabova et al., 2014[25]; Toth et al., 2020[60]). They are characteristically protein in nature. Thus, pathologic intolerance or physiological tolerance to them during prolonged chronic use could arise due to increased clearance by the reticuloendothelial system or induction of hypersensitivity by the adaptive immune system, respectively (Ochin and Garelnabi, 2018[41]). Given all these, specific nutraceuticals might be worth considering as supportive treatments for lipid-lowering (Banach et al., 2018[6]; Fan et al., 2021[22]; Ochin and Garelnabi, 2018[41]). In several previous studies, traditional Chinese medicine has been demonstrated as a strategy to prevent and treat atherosclerosis (Ting-Ting et al., 2019[59]; Zhang et al., 2019[65]).

## What is Berberine, and How Does it Lower Cholesterol?

Berberine (BBR, C_20_H_18_NO_4_^+^) is a quaternary ammonium salt derived from isoquinoline alkaloids, widely used in Ayurvedic and Chinese medicine due to its favorable clinical and safety profile (Jin et al., 2016[30]; Ju et al., 2018[31]; Lin and Zhang, 2018[36]; Tillhon et al., 2012[58]). It belongs to the class of protoberberines found in several plants, including *Berberis vulgaris*, *Coptis Chinensis*, and *Berberis aristata*, or obtained through total synthesis (Huang et al., 2011[26]; Imanshahidi and Hosseinzadeh, 2008[27]; Kong et al., 2004[34]). Considering the latest advances in pharmacological research, BBR appears to be one of the most promising natural product-derived drugs for treating cardiovascular, renal, and metabolic diseases (CVMDs) (Feng et al., 2019[23]; Bagherniya et al., 2018[4]; Talebi et al., 2020[57]; Yaribeygi et al., 2021[63]). The natural cholesterol-lowering agent, berberine, has been found to have antidyslipidemic properties (Kong et al., 2004[34]). It can lower both adipogenesis and lipid synthesis (Choi et al., 2006[11]). Berberine directly facilitates the stabilization of LDLR mRNA by activating regulatory proteins downstream of the extracellular signal-regulated kinase (ERK) pathway; those regulatory proteins interact with the proximal sequences in the 3′ untranslated region (UTR) of LDLR mRNA, and by activating Jun amino-terminal kinase (JNK)-dependent pathways (Abidi et al., 2005[1]; Li et al., 2009[35]). In addition, berberine has demonstrated anti-PCSK9 effects by ubiquitination and degradation of hepatocyte nuclear factor 1 alpha (HNF1α), an essential cofactor for sterol regulatory element-binding protein-2 (SREBP-2) in PCSK9 transcriptional regulation (Li et al., 2009[35]; Shafabakhsh et al., 2021[53]). Studies have demonstrated that berberine is a trans-activator of PCSK9 expression and inhibits its transcription via the HNF1 binding site of the PCSK9 promotor (Shafabakhsh et al., 2021[53]). Berberine improved HNF1α protein degradation as well, leading to promoting the expression of LDLR and limiting its degradation, resulting in greater clearance of LDL (Dong et al., 2015[21]; Fan et al., 2021[22]; Ochin and Garelnabi, 2018[41]) (Figure 1b(Fig. 1)). Many studies have shown berberine's effect on PCSK9 expression, suggesting PCSK9 may be a target for berberine (Ochin and Garelnabi, 2018[41]). In the following section, we discuss several *in vitro *and* in vivo* studies showing berberine-mediated modulation of PCSK9 mRNA expression, protein level, and function (Tables 1-3(Tab. 1)(Tab. 2)(Tab. 3); References in Table 1: Cameron et al., 2008[9]; Dong et al., 2015[21]; Jia et al., 2014[29]; Li et al., 2009[35]; Liu et al., 2015[37]; Ochin and Garelnabi, 2018[41]; Vahedian-Azimi et al., 2021[61]; Xiao et al., 2012[62]; References in Table 2: Cameron et al., 2008[9]; Formisano et al., 2020[24]; Li et al., 2009[35]; Pisciotta et al., 2012[43]; Spigoni et al., 2017[55]; References in Table 3: Liu et al., 2015[37]; Fan et al., 2021[22]).

## Cell Culture and Animal Studies

In a study by Cameron et al., berberine (44 µM) was shown to decrease PCSK9 mRNA by 77 % and PCSK9 protein levels by 87 % in HepG2 cells, which was associated with a 3-fold increase in LDLR mRNA expression (Cameron et al., 2008[9]). They reported that this reduction was most likely caused by a decrease in PCSK9 gene transcription rather than an increase in PCSK9 mRNA degradation. Additionally, when HepG2 cells were simultaneously treated with berberine and mevastatin, the effect of mevastatin on PCSK9 mRNA was suppressed, and the amount of PCSK9 mRNA was decreased by 56 %; therefore, the LDLR-rising effect was enhanced. Compared with cells receiving mevastatin alone, there was a 1.4-fold rise in LDLR protein levels and a 6-fold increase in LDLR mRNA (Cameron et al., 2008[9]). Similarly, in another study by Li et al. on HepG2 cells, 20 μM of berberine treatment decreased PCSK9 mRNA expression (Li et al., 2009[35]). They detected a 30 % reduction at 12 h and a gradual decline in the mRNA level of PCSK9 in berberine-treated cells down to 23 % of untreated control by 48 h. On the other hand, they detected an increase in LDLR mRNA expression by 1.8-fold at four h and up to 3-fold at 24 h (Li et al., 2009[35]). Moreover, they treated HepG2 cells with 20 μM or 40 μM of berberine in the absence or presence of 1 μM of fluvastatin, lovastatin, or simvastatin for 24 h and found that although statins independently increased PCSK9 mRNA levels above 2-fold of control, their inducing effects were not observed in cells simultaneously treated with berberine at both mentioned doses (Li et al., 2009[35]). It also has been reported by Xiao et al. that administration of 10 or 30 mg/kg/day of berberine for four weeks prevented the induction of PCSK9 mRNA expression, attenuation of LDLR mRNA, and elevation of plasma LDL-C in C57BL/6 mice who were co-treated with 5 mg/kg/day LPS (Xiao et al., 2012[62]). Nevertheless, in another study, Jia et al. examined the effects of berberine administration (400 mg/kg/day) for six weeks in HFD rats and obtained inconsistent results (Jia et al., 2014[29]). They showed that berberine dramatically raises plasma levels of PCSK9. It raised the mRNA and protein expression levels of LDLR in the liver and lowered plasma LDL-C concentrations in HFD rats compared to control rats (Jia et al., 2014[29]). Genetic polymorphisms may cause such variations in PCSK9 responsiveness to berberine in the PCSK9 and LDLR promoters, affecting how berberine interacts with the promoter and ultimately changing gene transcription (De Castro-Oros et al., 2016[17]).

Liu et al. examined the effects of berberine (156 mg/kg/day) and 8-hydroxy-dihydro-berberine (Hdber) (78, 39, and 19.5 mg/kg/day), a structurally modified version of berberine, on HFD rats (Liu et al., 2015[37]). The two compounds markedly lowered the expression levels of PCSK-9 protein, associated with elevated expression levels of LDL-R protein and a subsequent decline in serum LDL-C (total cholesterol) TC, and triglyceride (TG). In addition, rats in BBR and those treated with high doses of hdber (78 mg/kg/day) displayed significantly elevated HDL-C levels in their serum. They indicated that hdber could make similar effects as berberine when only 25 % of the original dosage of berberine needed were administered (Liu et al., 2015[37]).

Dong et al., in another study, evaluated PCSK9 levels and hepatic LDLR expression changes in HFD mice and hamsters treated with 200 mg/kg/day and 100 mg/kg/day berberine, respectively (Dong et al., 2015[21]). After 16 days of treatment with berberine, serum PCSK9 levels and its hepatic mRNA expression were reduced by 50 % and 46 %, respectively, in HFD mice; leading to increased protein levels of hepatic LDLR (67 %) and lowered serum cholesterol levels (TC and LDL-C) in HFD mice compared to controls (Dong et al., 2015[21]). Furthermore, they evaluated the effect of berberine treatment on HFD hamsters with 100 mg/kg/day of berberine over seven days to evaluate its efficacy in other animal models. The hamsters in the treated group showed a reduction of 30 % in PCSK9 serum levels compared to the control group (Dong et al., 2015[21]). Consistent with the results mentioned above, they reported significant reductions in mRNA and protein expression of PCSK9 levels in HepG2 cells after 16 and 24 h of berberine treatment (40 μM) (Dong et al., 2015[21]).

In 2018, Ochin and Garelnabi published observations indicating an association between berberine release from PLGA-PEG-PLGA encapsulation polymer and the expression of PCSK9 mRNA and protein in a time-dependent manner (Ochin and Garelnabi, 2018[41]). They demonstrated a significant downregulation of PCSK9 mRNA in HepG2 cells treated with 150 μM (high-FD) and 10 μM (low-FD) concentrations of free berberine drug solution and 150 μM of berberine encapsulated nanoparticles in PVA surfactant nanoemulsion (BC-NP) during a 24-hour and 48-hour period (Ochin and Garelnabi, 2018[41]). They showed that the cells treated with low FD showed the highest reduction in PCSK9 mRNA at the end of the 24-hour treatment period, closely followed by the cells treated with high-FD, and ultimately by the cells treated with BC-NP, while the untreated cells showed the most significant expression of PCSK9 mRNA (Ochin and Garelnabi, 2018[41]). However, at the end of 48 hours, PCSK9 mRNA expression was significantly lower in cells treated with high-FD than in those treated with low-FD, whose levels had already risen (Ochin and Garelnabi, 2018[41]). Compared to the same group of cells from the 24-hour treatment duration, the cells treated with BC-NP displayed a considerable reduction in PCSK9 mRNA, with levels almost similar to the low-FD cells from the 48-hour treatment duration (Ochin and Garelnabi, 2018[41]). Moreover, the 24-hour treatment period revealed that Hep-G2 cells in the low concentration group of FD (FD-low) expressed PCSK9 protein at the lowest levels, this were followed by the expression level in the FD-high concentration group, and the BC-NP group has an expression level close to what the cells in the untreated group synthesize (Ochin and Garelnabi, 2018[41]). Nevertheless, the 48-hour treatment period with equal berberine concentrations and forms demonstrates that PCSK9 expression levels at low and high concentrations of FD remain the same. On the other hand, the PCSK9 expression level in the BC-NP treated cell group was significantly decreased (Ochin and Garelnabi, 2018[41]). In addition, after 48 hours of BC-NP, low-FD, or high-FD treatment with the same berberine concentrations, the authors also found a significant upregulation of LDLR mRNA expression (Ochin and Garelnabi, 2018[41]).

In one of the most recent research, Fan et al. synthesized 40 berberine derivatives and evaluated them for their ability to suppress PCSK9 transcription in HepG2 cells, with berberine serving as the benchmark (Fan et al., 2021[22]). Analysis of the structure-activity relationship suggested that the 2,3-dimethoxy moiety would favor activity. The most powerful activity among them, superior to berberine's, was shown by 9k (Fan et al., 2021[22]). Compound 9K (5 and 10 μM) significantly decreased the level of PCSK9 protein in hepG2 cells. 9k administrating (50 mg/kg) every day for four weeks also decreased PCSK9 protein levels in the liver and serum of C57BL/6J mice (Fan et al., 2021[22]). Furthermore, they evaluated the effects of 9k on LDLR expression and LDL-C uptake in HepG2 cells (Fan et al., 2021[22]). They showed that 9k treatment significantly upregulated LDLR protein levels in a dose-dependent manner. Moreover, compound 9k (20 μM) markedly promoted DiI-LDL uptake in a dose-dependent manner (5, 10, 20 μM) with a scale of 2-3 folds, which was much more effective than that of BBR (20 μM) (Fan et al., 2021[22]).

Ma et al. (2021[38]) published results indicating that BBR treatment at doses of 50 and 100 mg/kg/d could enhance lipid metabolism in the serum by significantly lowering total cholesterol (TC), triglyceride (TG), low-density lipoprotein (LDL-C), and increasing high-density lipoprotein cholesterol (HDL-C) levels in ApoE-/- mice fed with HFD. Berberine could decrease PCSK9 mRNA and hepatic protein levels and increase LDLR mRNA and protein in the liver of ApoE-/- mice fed with HFD (Vahedian-Azimi et al., 2021[61]). They also showed that berberine at the mentioned doses decreased aorta atherosclerotic plaque and ameliorated lipid deposits in ApoE-/- mice fed with HFD (Vahedian-Azimi et al., 2021[61]). Moreover, BBR (0, 5, 25, and 50 μg/mL) treatment of HepG2 cells led to increased LDLR protein expression and decreased PCSK9 protein expression through the extracellular signal-regulated kinase 1/2 (ERK1/2) pathway (Vahedian-Azimi et al., 2021[61]).

## Clinical Studies

A clinical trial conducted by Pisciotta et al. investigated the impact of a nutraceutical pill containing berberine (500 mg), policosanol (10 mg) , and red yeast rice (200 mg) in subjects with primary hypercholesterolemia (HCH) with a history of statins (STs) intolerance or refusing STs treatment (Pisciotta et al., 2012[43]). Nutraceutical pill was found to lower LDL-C by 31.7 % after six months of follow-up, which was more efficacious and better tolerated than ezetimibe (EZE) (Pisciotta et al., 2012[43]). In the same study, supplementary treatment with nutraceutical pills in heterozygous hypercholesterolemia (HeFH) patients on stable-dose treatment with STs or STs plus EZE resulted in a greater reduction of LDL-C (mean reduction of 10.5 %). The reduction (37.1 %) was even higher than doubling the dose of ST, and it was thought to be associated with an indirect, berberine-mediated inhibition of PCSK9 (Pisciotta et al., 2012[43]). 

A 12-week treatment with a nutraceutical formulation containing putative lipid-lowering compounds (berberine 200 mg, chitosan, red yeast rice) was effective in lowering plasma non-HDL-C (by ~15 %) and LDL-C (by 20 %) compared with placebo, according to a double-blind, randomized, placebo-controlled study (Spigoni et al., 2017[55]). Notably, during the study, the PCSK9 plasma levels remained stable in individuals with hypercholesterolemia (Spigoni et al., 2017[55]). Besides, three adverse effects were noted in the trial mentioned above, including duodenitis, Epstein-Barr virus infection, and an asymptomatic but considerable rise in creatine phosphokinase (after intense exercise) that necessitated hospitalization. Nevertheless, the intervention was well tolerated (Spigoni et al., 2017[55]).

A combination of a nutraceutical with monacolin K (MonK)+KA (1:1), berberine (500 mg), and silymarin proved effective as a lipid-lowering agent with a large interindividual response variability, according to a pilot trial (Formisano et al., 2020[24]). The result was comparable to what 10 mg of atorvastatin would have produced (Formisano et al., 2020[24]). Besides, eight weeks of nutraceutical administration resulted in a significant +25.6 % increase in serum PCSK9. They suggested that BBR activity may only partially suppress the PCSK9 increase when more bioavailable (Formisano et al., 2020[24]). Ultimately, NUT administration inhibited serum-mediated foam cell formation, as shown in human macrophages (Formisano et al., 2020[24]). The functional profile of lipoproteins with a probable antiatherogenic effect was improved by *ex vivo* NUT treatment (Formisano et al., 2020[24]).

## Conclusion

Berberine and berberine-like compounds have shown LDL-lowering effects in several studies and could significantly inhibit PCSK9 at both transcriptional and protein levels. Nevertheless, it must be noted that berberine cannot replace standard lipid-lowering therapy but may adjunctively help enhancing the activity of current therapies in reducing CV risk (Cicero et al., 2017[12]). Combining berberine with other lipid-lowering nutraceuticals could reduce the required dosages of single components, leading to safety improvement. Berberine can be a beneficial adjunct to statin therapy since the latter is accompanied by an elevation of PCSK9 levels, which limit the efficacy of statins. It is, however, necessary to conduct high-quality randomized controlled trials to ensure berberine's long-term efficacy in reducing CVD mortality and morbidity. 

## Conflict of interest

The authors declare no conflict of interest.

## Figures and Tables

**Table 1:**
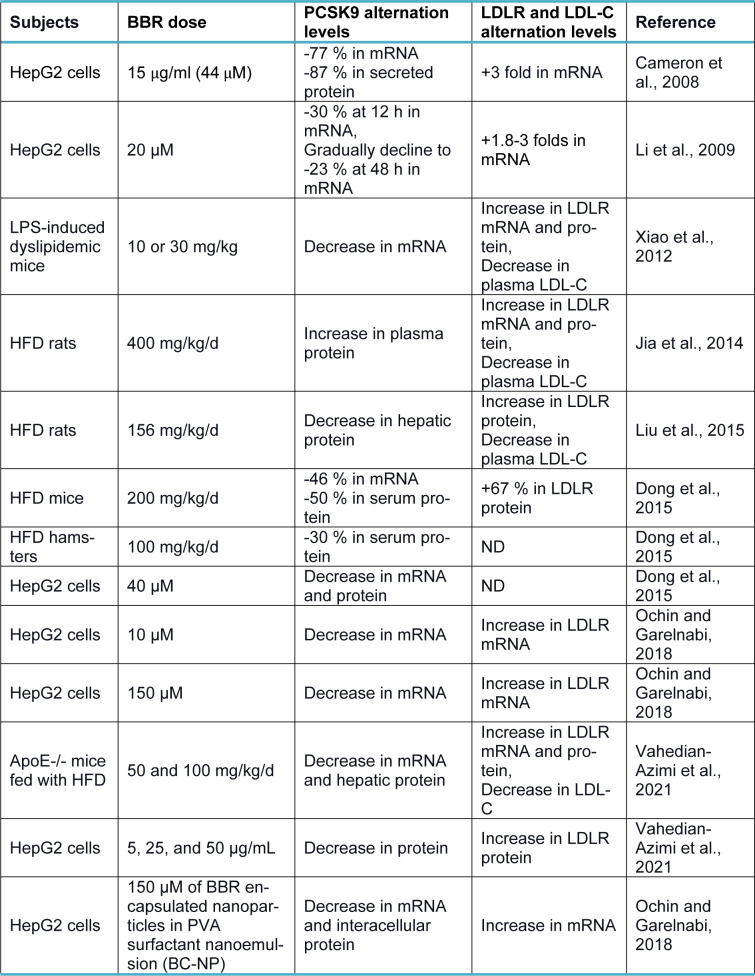
Effect of berberine on PCSK9, LDLR, and LDL-C levels in experimental studies

**Table 2:**
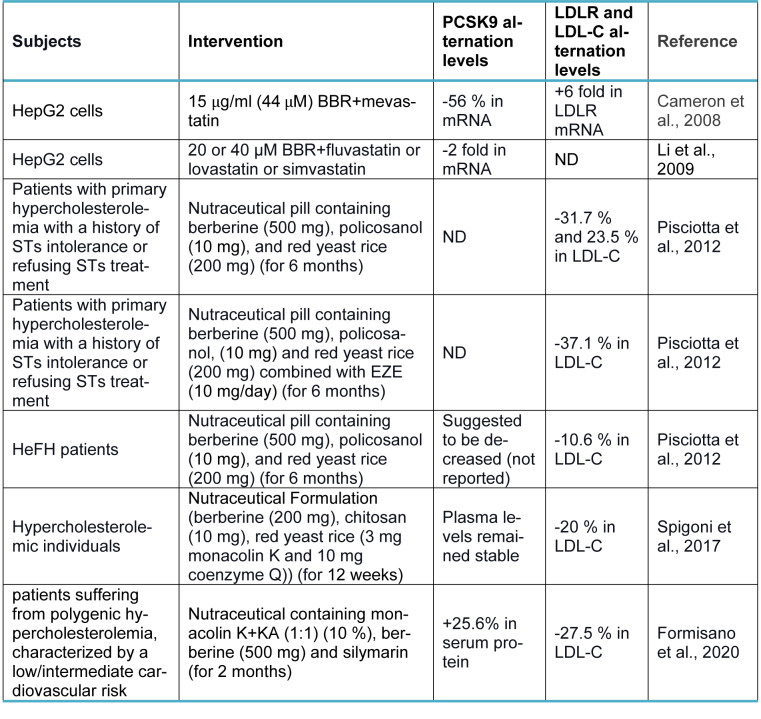
Effect of berberine on PCSK9, LDLR, and LDL-C levels in combination with other nutraceuticals or medications in experimental studies

**Table 3:**
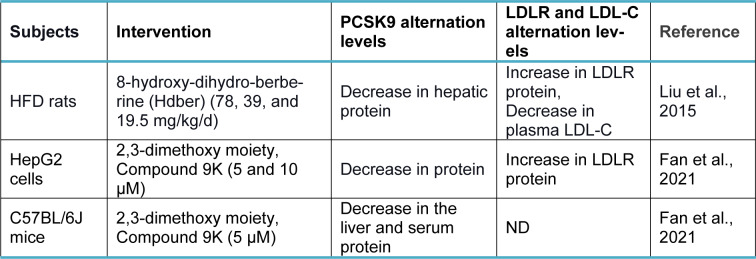
Effect of berberine derivatives and modified forms on PCSK9, LDLR, and LDL-C levels in experimental studies
